# Supplementary material for: SNP and Haplotype Regional Heritability Mapping (SNHap-RHM): Joint Mapping of Common and Rare Variation Affecting Complex Traits
Source: Front Genet. 2022 Jan 6;12:791712. doi: 10.3389/fgene.2021.791712 (PMC8770330; doi:10.3389/fgene.2021.791712)
Supplement: Supplementary file 1 [file DataSheet1.PDF]

Supplementary Material

1. Supplementary Text. Investigating the SNP-RHM and Hap-RHM with simulated phenotypes

1.1. LRT vs Region size

The LRT statistics decayed with increasing region size (when measuring size as number of markers in the region) for the two models even when the analysis models (SNP-RHM or Hap-RHM) matched the genetic architecture of the simulated phenotypes (Supplementary Figures 3 and 4). The rate of decay was more pronounced for Hap-RHM, in which the LRT had a non-linear relationship with the region size (Supplementary Figure 4).

1.2. LRT vs Variance estimates

For the SNP QTL phenotypes, the variance estimated by the SNP-RHM in regions where we simulated effects was significantly different from zero (except two regions in both phenotypes) (Supplementary Figure 5). The plots show strongly positive curvilinear relationship between the LRTs and the estimated variance. As the LRT increases, the variance approaches and then crosses the simulated variance. This is to be expected and is true for real data as well (e.g., the winner's curse is a similar phenomenon).

On the other hand, the relationship between the LRT and regional variance estimates is less clear for the haplotype QTL phenotypes (Supplementary Figure 6). The plots, again, show a non-linear relationship, and once the LRT passes the Bonferroni-corrected threshold for genome-wide significance (red horizontal dashed lines), the variance estimates approached the simulated value of 0.005 (blue vertical line).

1.3. Region size vs Variance estimates

Supplementary Figure 7 shows a curvilinear relationship, albeit not significant, between number of markers in the region and the estimated regional variance for the SNP QTL phenotypes. However, the relationship is very strong for the haplotype QTL phenotypes (Supplementary Figure 8). The estimated regional variances are inflated when a region has more than 5,000 different haplotype alleles. These regions harbour lots of rare haplotypes, which means the haplotypes chosen as QTLs in the simulation are more likely to be rare. Therefore, the inflation may be an artefact of estimation procedure, which indirectly assumes that rare variants with lower MAF will have larger allele effects and thus by design overestimates their effect. This happens because the GREML model normalises the marker genotypes and assumes that the effect size per normalised genotype follows a normal distribution.

1.4. LRT vs Allele frequencies

We further investigated whether the allele frequencies of the QTLs influence the LRT statistics and the estimated regional variances. We show in Supplementary Figure 10 that there is no relationship between allele frequencies of the SNP QTLs and LRTs. There is, however, a linear and curvilinear relationship between LRTs and the haplotype frequencies of the rare and common haplotype QTLs, respectively. The haplotypes simulated to have an effect in regions with an overestimated regional variance are marked with different colours on these plots. These haplotypes are shown to have rare haplotype frequencies and relatively low LRTs (Supplementary Figure 10).

These results also show that the QTLs for the 1-SNP QTL phenotypes and one common haplotype QTL phenotypes are evenly distributed across the MAF spectrum, whereas the QTLs for the one rare haplotype QTL phenotypes have a MAF distribution that is skewed towards zero.

2. Supplementary Figures and Tables

2.1. Supplementary Figures

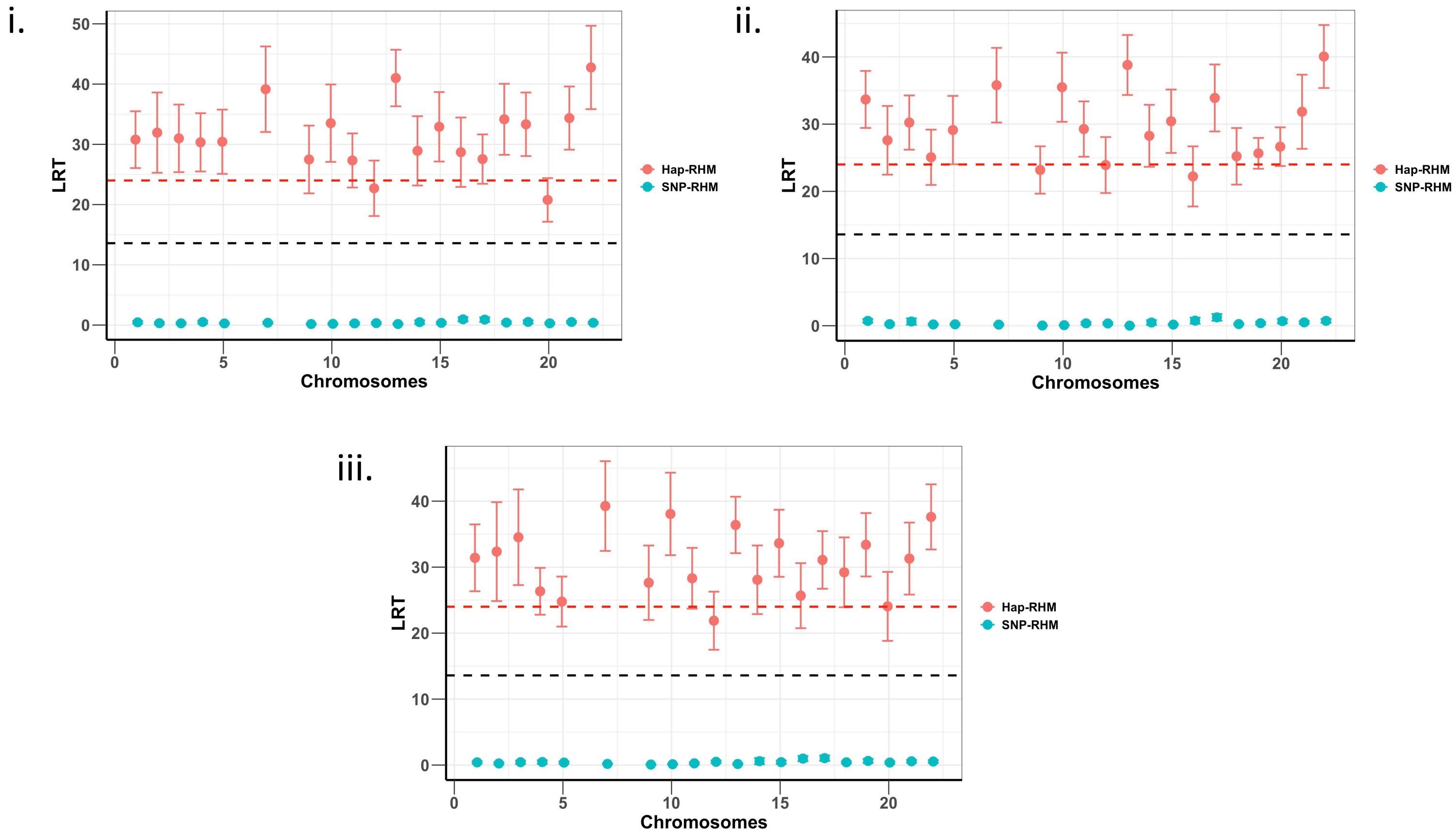

Supplementary Figure 1. Plots of average LRT statistics over replicates of QTL loci across the chromosomes for the 20 simulations of each of the three haplotype QTL phenotypes. The red dashed lines are genome-wide significance threshold (for 48,772 regions) and the black dashed lines are Bonferroni significance threshold (for 220 regions). The plot (i) is the 1-rare haplotype QTL phenotype, the plot (ii) is the 1-common haplotype QTL phenotype, and the plot (iii) is the multiple haplotype QTL phenotype. The three phenotypes are analysed using both the SNP based model (SNP-RHM) (blue points) and the Haplotype based model (Hap-RHM) (red points)The SNP-RHM fails to capture the simulated effects for the haplotype QTLs.

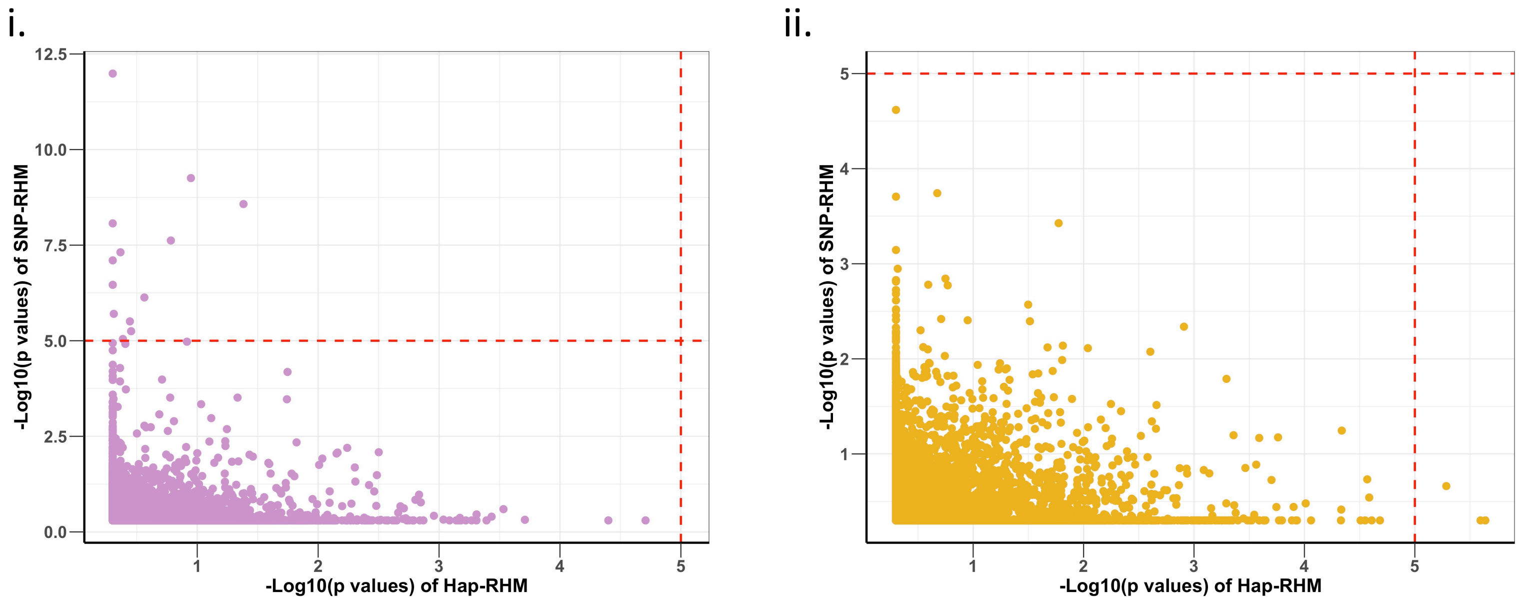

Supplementary Figure 2. The two analysis models (SNP-RHM and Hap-RHM) are independent of each other in the analysis of i. height, and ii. Major depressive disorder. The minus log10 of p values of association of regions for the SNP-RHM are plotted against that of the Hap-RHM. The red vertical and horizontal dashed lines are the suggestive significance threshold at p-value $< 1 \times 10^{-5}$. The plots show that different significant regions are identified by each model for both phenotypes. The correlation of the regional associations between the two models are 0.11 for height and 0.16 for MDD.

i.

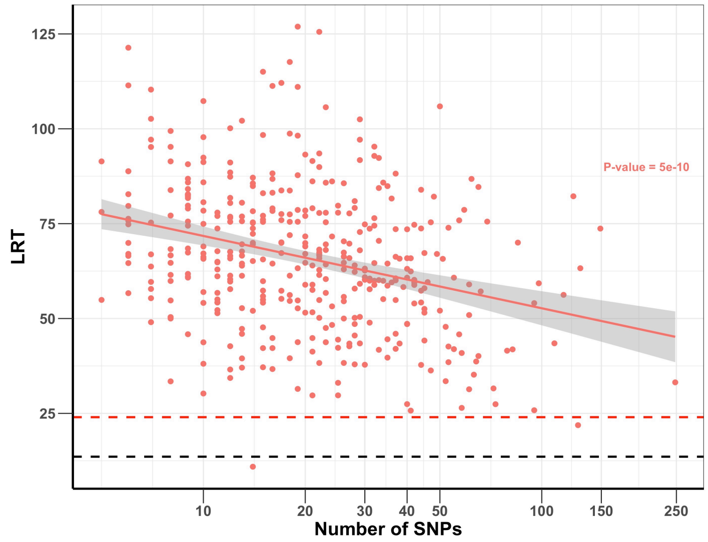

ii.

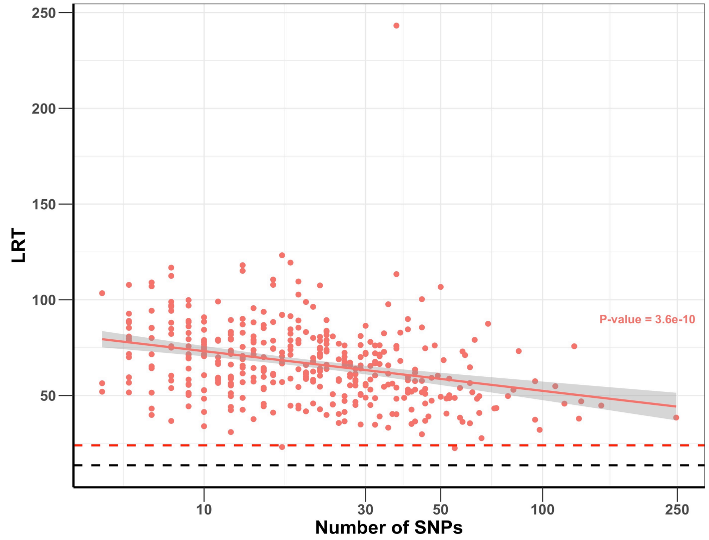

Supplementary Figure 3. Plots of LRT statistics against QTL region size for the 20 simulations (not averaged) of each of the two SNP QTL phenotypes. The red dashed lines are genome-wide significance threshold (for 48,772 regions) and the black dashed lines are Bonferroni significance threshold (for 220 regions). Plot (i) is the 1-SNP QTL phenotype, and the lower plot (ii) is the multiple SNP QTL phenotype. In both phenotypes the LRT statistic reduced with increasing region size.

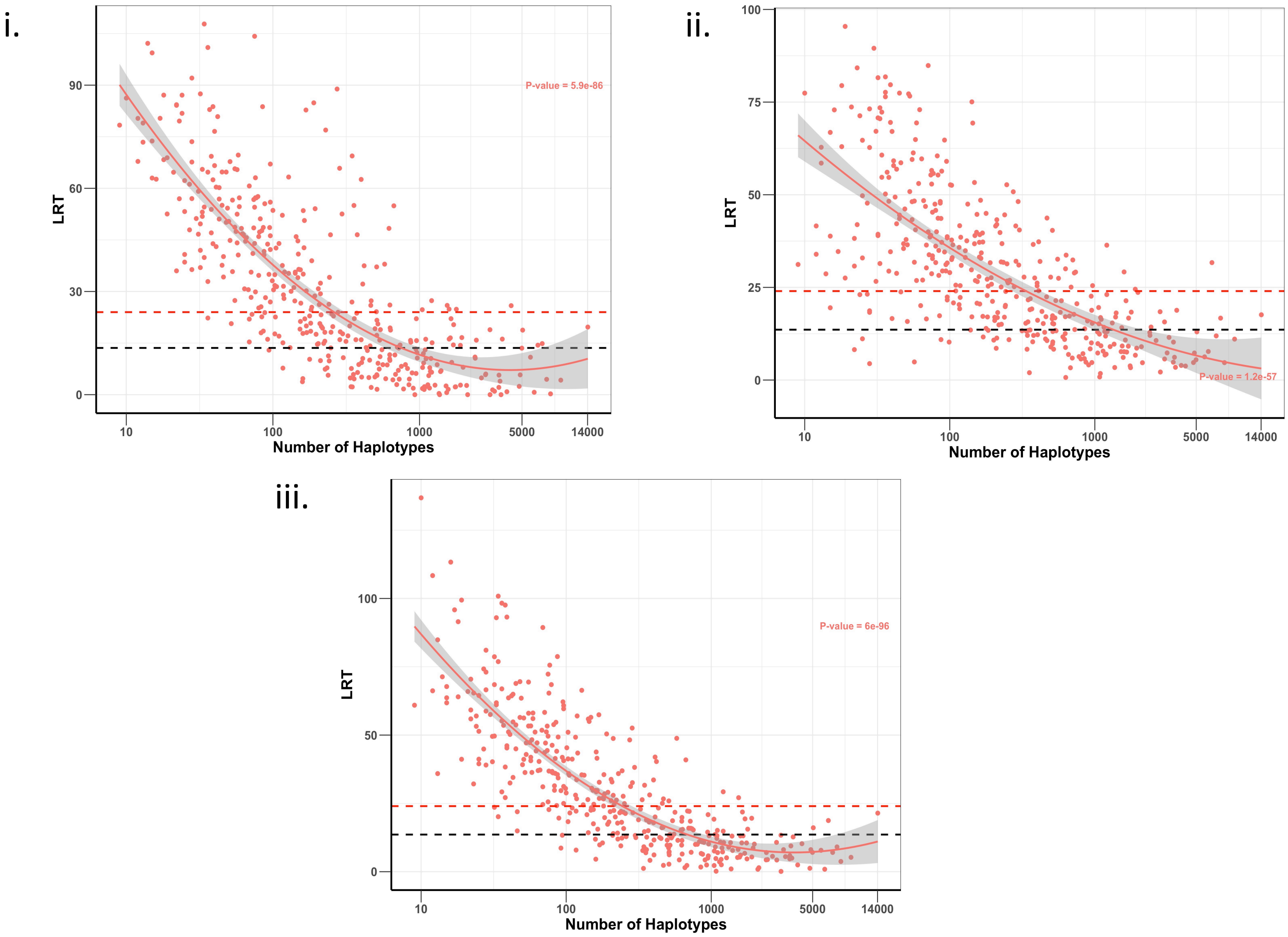

Supplementary Figure 4. Plots of LRT statistic against QTL region size for the 20 simulations of each of the three haplotype QTL phenotypes. The red dashed lines are genome-wide significance threshold (for 48,772 regions) and the black dashed lines are Bonferroni significance threshold (for 220 regions). The plot (i) is the 1-rare haplotype QTL phenotype, the plot (ii) is the 1-common haplotype QTL phenotype, and the plot (iii) is the multiple haplotype QTL phenotype. For all the three phenotypes the LRT statistic reduced with increasing region size.

i.

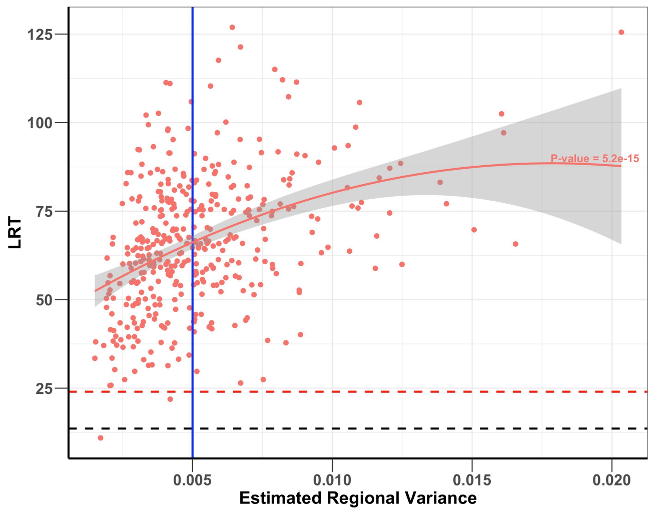

ii.

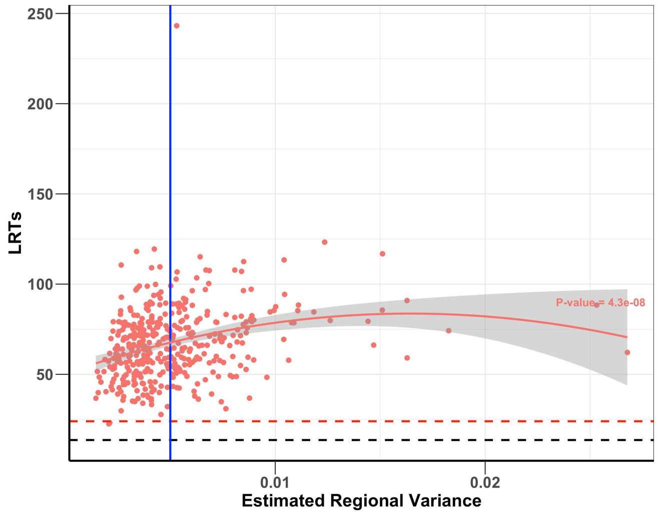

Supplementary Figure 5. Plots of LRT statistic against estimated regional variance for the 20 simulations of the single SNP QTL phenotype. The red dashed lines are genome-wide significance threshold (for 48,772 regions), the black dashed lines are Bonferroni significance threshold (for 220 regions), and the blue vertical line is the simulated regional variance of 0.005. The plot (i) is the 1 – SNP QTL phenotype, and the lower plot (ii) is the multiple SNP QTL phenotype. The estimated regional variance clustered closely around the simulated value for most of the regions. The variances of a few regions were overestimated in both phenotypes.

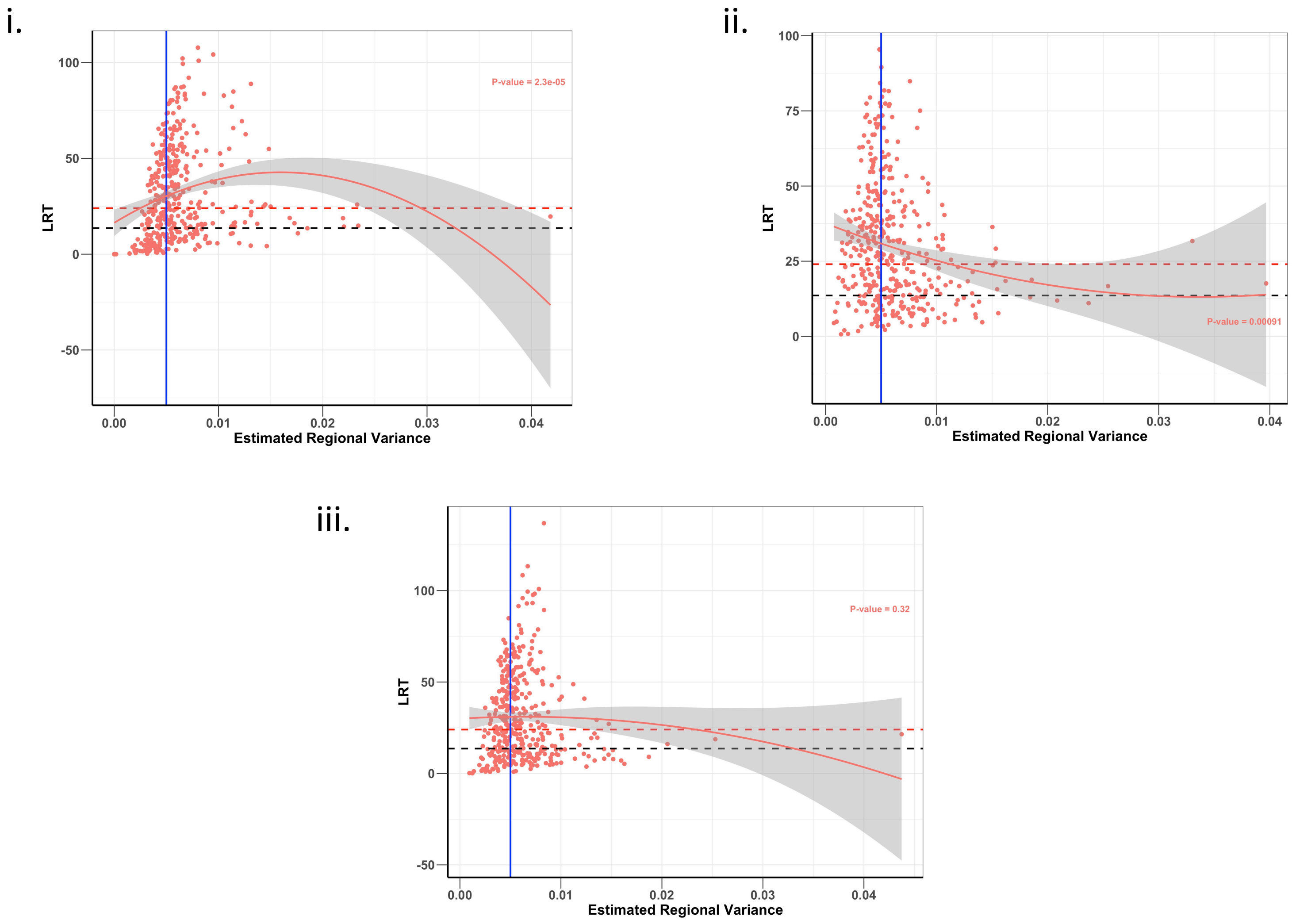

Supplementary Figure 6. Plots of LRT statistic against estimated regional variance for the 20 simulations of each of the three haplotype QTL phenotypes. The red dashed lines are genome-wide significance threshold (for 48,772 regions), the black dashed lines are Bonferroni significance threshold (for 220 regions), and the blue vertical line is the simulated regional variance of 0.005. The plot (i) is the 1-rare haplotype QTL phenotype, the plot (ii) is the 1-common haplotype QTL phenotype, and the plot (iii) is the multiple haplotype QTL phenotype. The estimated regional variance clusters closely around the simulated value for the regions that pass the Bonferroni threshold.

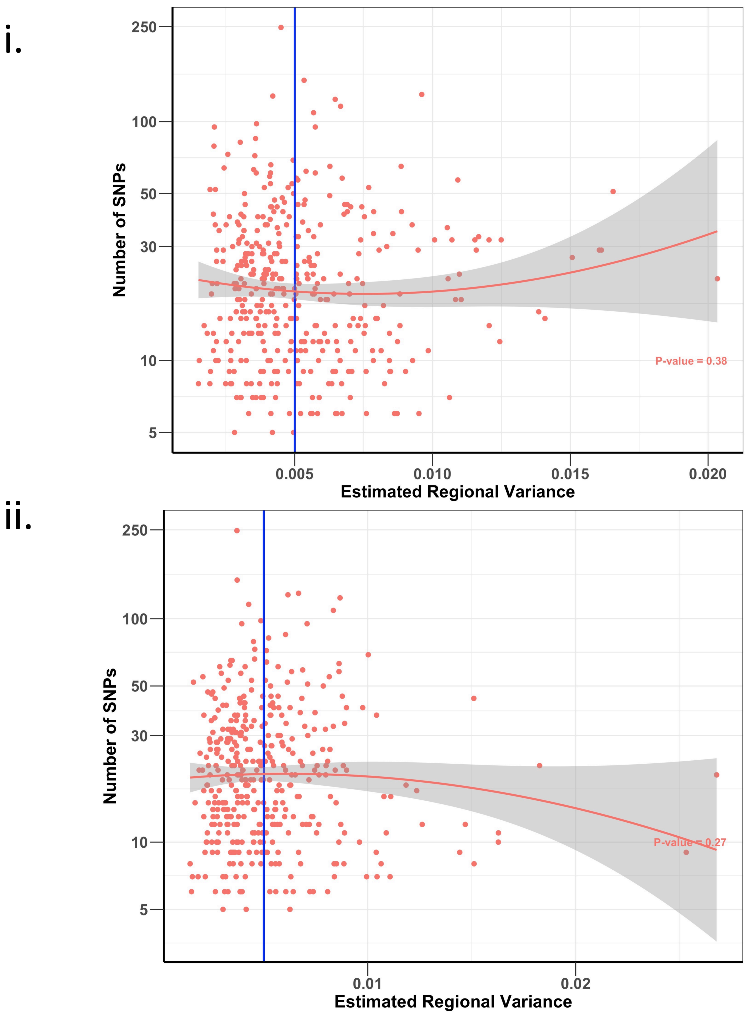

Supplementary Figure 7. Plots of region size against estimated regional variance for the 20 simulations of the two SNP QTL phenotype. The blue vertical line is the simulated regional variance of 0.005. The plot (i) is the 1-SNP QTL phenotype, and the lower plot (ii) is the multiple SNP QTL phenotype. The two plots show there is no significant relationship between estimated regional variance and region size.

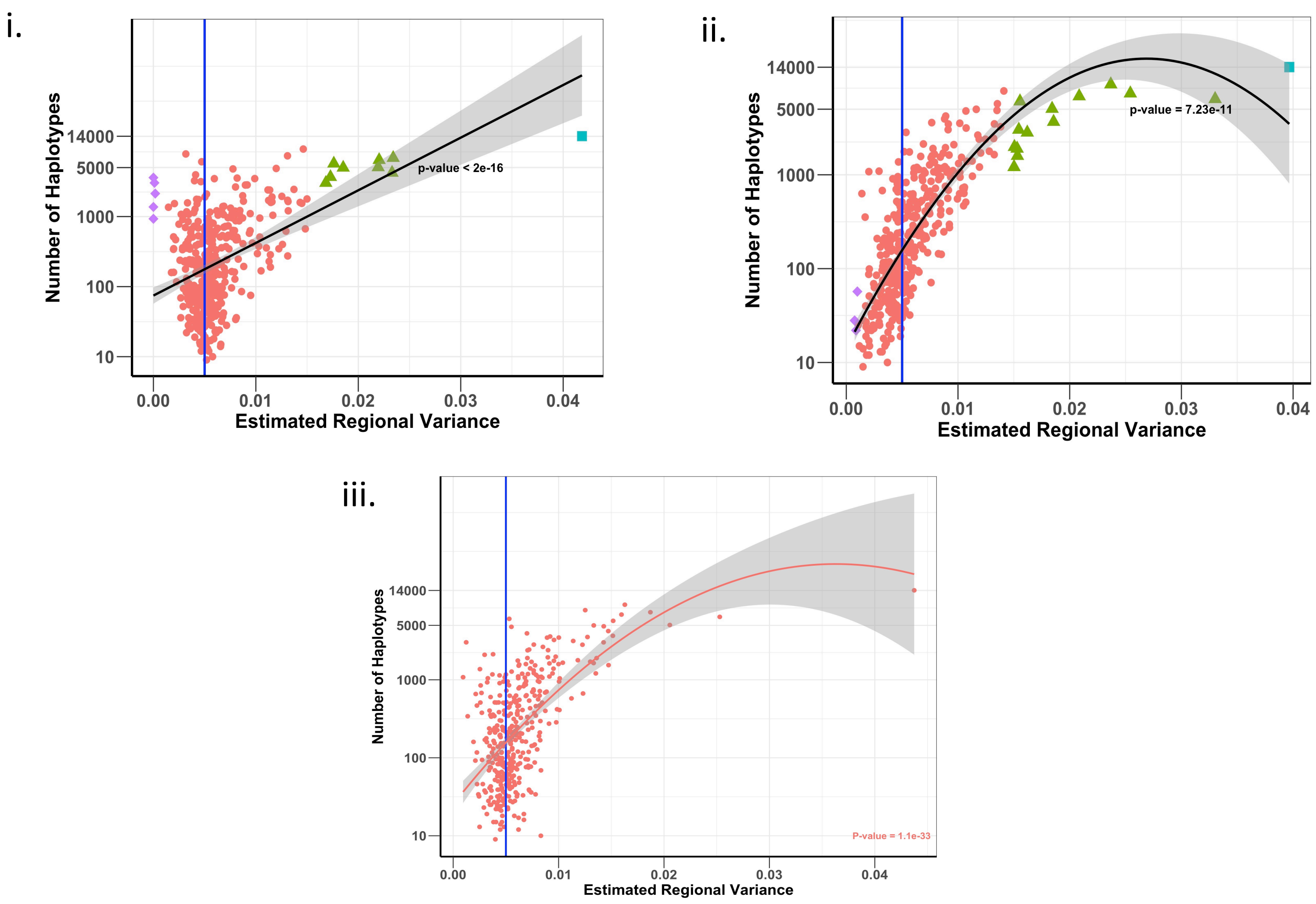

Supplementary Figure 8. Plots of region size against estimated regional variance for the 20 simulations of the three haplotype QTL phenotype. The blue vertical line is the simulated regional variance of 0.005. The plot (i) is the 1-rare haplotype QTL phenotype, and the plot (ii) is the 1-common haplotype QTL phenotype. On these plots, the blue square point is the region with the largest overestimated variance, the green triangle points are regions with overestimated variance, red points are all other regions and purple points are regions with least variance estimates. The plot (iii) is the multiple haplotype QTL phenotype. The plots show that the estimates of the regional variances are likely to be inflated when the region size gets beyond 1000 haplotypes

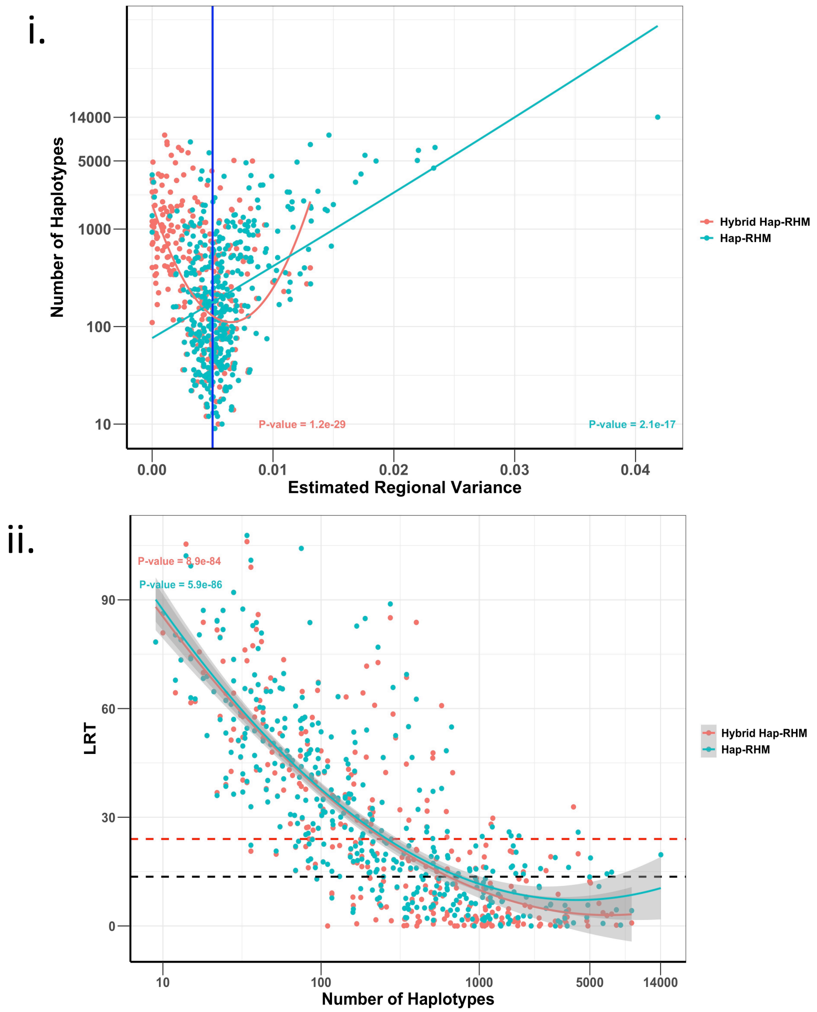

Supplementary Figure 9. Plots for the 1-rare haplotype QTL phenotype analysed using Hap-RHM (red points) and a hybrid variant of the Hap-RHM (blue points). The hybrid Hap-RHM broke larger regions (regions with more than 20 SNPs) into smaller regions of 20 or less SNPs and used that to determine the haplotypes. Each blue point represents the estimated variances or LRT for best sub-window within the bigger window. (i) is a plot of region size over estimated regional variance for the 20 simulations of the phenotype and (ii) is the plot of LRT statistic over QTL region size for the 20 simulations of each of the phenotype. The plot (i) shows that the hybrid Hap-RHM underestimates the regional variance at larger regions. The LRT statistics however do not improve very much over the default Hap-RHM.

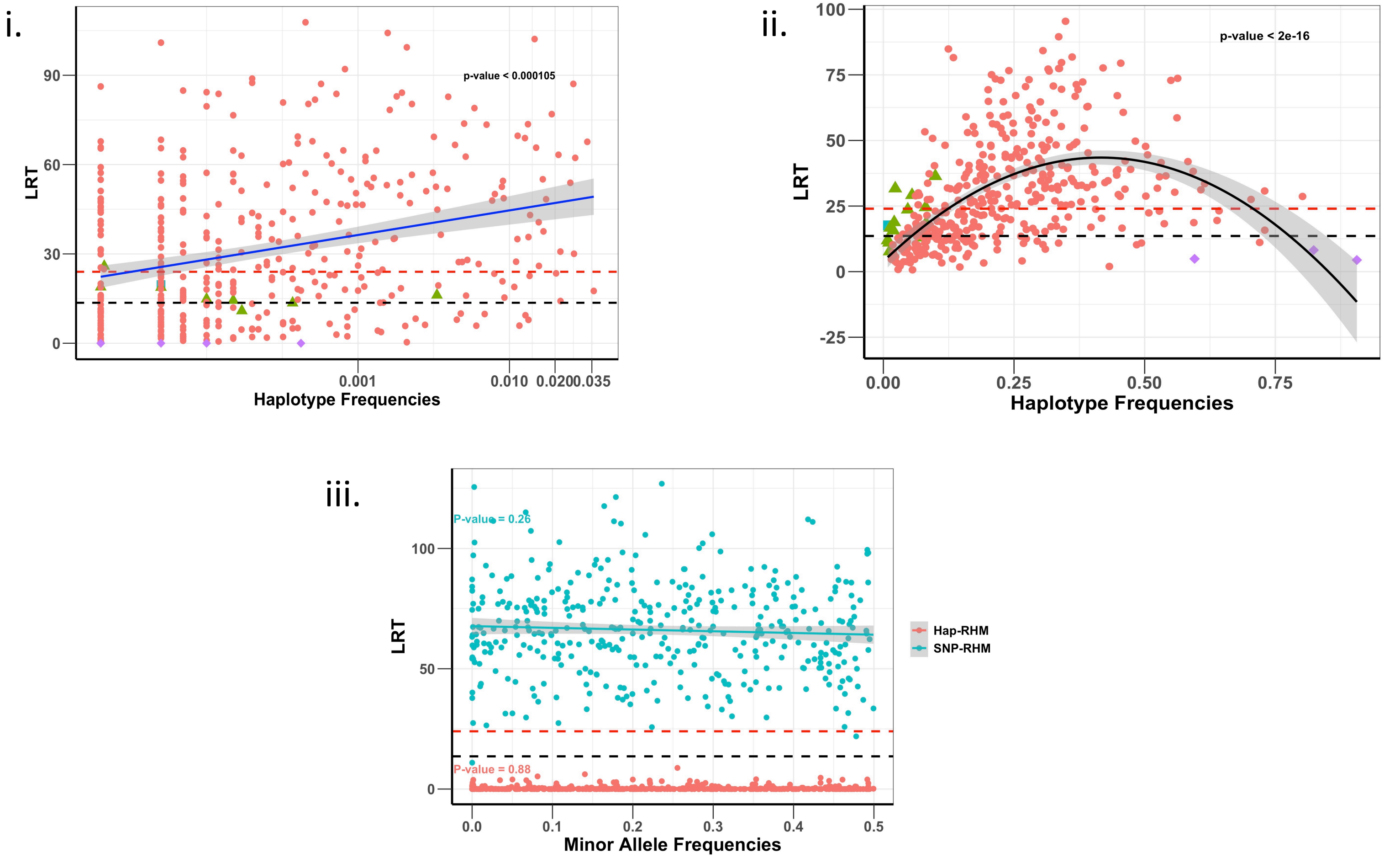

Supplementary Figure 10. Plots of LRT statistic against QTL marker frequencies. The red dashed lines are genome-wide significance threshold (for 48,772 regions) and the black dashed lines are Bonferroni significance threshold (for 220 regions). The plots (i) and (ii) are 1 rare and 1 common haplotype QTL phenotypes, respectively. On these plots, the blue square point is the region with the largest overestimated variance, the green triangle points are regions with overestimated variance, red points are all other regions and purple points are regions with least variance estimates. The plot (iii) is the 1-SNP QTL phenotype analysed using SNP-RHM (blue points) and Hap-RHM (red points). The three plots show that there is no relationship between QTL marker frequencies and the LRT statistic.

2.2. Supplementary Tables

Supplementary Table 1. Top genomic regions identified by SNP/ haplotype-based model for Height. The columns are chromosome number, the association p-values, the genes within region, genes within 400kb of regions, associated trait, and a list of studies reporting gene association with trait.

SNP based regional GREML model									
CHR	P value	Genes	Genes within 400kb	Trait	Reference Study				
					PMID	First author	Journal	Discovery sample (size and ancestry)	Replication sample (size and ancestry)
1	1.78E-05		SPAG17	height	18391952	Weedon M N	Nat Genet	13,665 European ancestry individuals	16,482 European ancestry individuals
					20546612	Zhao J	BMC Med Genet	8,184 children of European ancestry	NA
2	4.86E-08	EFEMP1		height	20881960	Lango Allen H	Nature	133,653 European ancestry individuals	50,074 European ancestry individuals
					18391952	Weedon M N	Nat Genet	13,665 European ancestry individuals	16,482 European ancestry individuals
					25282103	Wood AR	Nat Genet	253,288 European ancestry individuals	80,067 European ancestry individuals
					31217584	Wojcik GL	Nature	17,286 African American individuals, 22,192 Hispanic/Latino individuals, 4,680 Asian ancestry individuals, 3,939 Native Hawaiian ancestry individuals, 647 Native American ancestry individuals,	NA
			MIR217, MIR216A	height	28552196	Tachmazidou I	Am J Hum Genet	53,588 European ancestry individuals	205,003 European ancestry individuals
			PNPT1	height	25429064	He M	Hum Mol Genet	36,227 East Asian ancestry individuals	57,699 East Asian ancestry individuals
					20546612	Zhao J	BMC Med Genet	8,184 children of European ancestry	NA
			SMEK2	height	30595370	Kichaev G	Am J Hum Gene	458,000 European ancestry individuals	NA
3	5.57E-10	ZBTB38		height	18391952	Weedon M N	Nat Genet	13,665 European ancestry individuals	16,482 European ancestry individuals
					25282103	Wood AR	Nat Genet	253,288 European ancestry individuals	80,067 European ancestry individuals
					23563607	Berndt SI	Nat Genet	8,097 European ancestry tall individuals, 8,099 European ancestry short individuals	4,872 European ancestry tall individuals, 4,831 European ancestry short individuals
		RASA2	RNF7	height	28552196	Tachmazidou I	Am J Hum Genet	53,588 European ancestry individuals	205,003 European ancestry individuals
			PXYLP1	height	28552196	Tachmazidou I	Am J Hum Genet	53,588 European ancestry individuals	205,003 European ancestry individuals
			GRK7	height	30595370	Kichaev G	Am J Hum Gene	458,000 European ancestry individuals	NA
4	3.12E-06	NCAPG		height	18391951	Gudbjartsson DF	Nat Genet	30,968 European ancestry individuals	8,541 European ancestry individuals
					20546612	Zhao J	BMC Med Genet	8,184 children of European ancestry	NA
		LCORL		height	22021425	Carty CL	Hum Mol Genet	8,149 African American female individuals	Up to 20,809 African American and African ancestry individuals
					20881960	Lango Allen H	Nature	133,653 European ancestry individuals	50,074 European ancestry individuals
					18391952	Weedon M N	Nat Genet	13,665 European ancestry individuals	16,482 European ancestry individuals

					25282103	Wood AR	Nat Genet	253,288 European ancestry individuals	80,067 European ancestry individuals
					28270201	Nagy R	Genome Med	19,920 British ancestry individuals from 6863 families.	NA
		DCAF16, FAM184B		height	31217584	Wojcik GL	Nature	17,286 African American individuals, 22,192 Hispanic/Latino individuals, 4,680 Asian ancestry individuals, 3,939 Native Hawaiian ancestry individuals, 647 Native American ancestry individuals,	NA
			MED28	height	30595370	Kichaev G	Am J Hum Gene	458,000 European ancestry individuals	NA
4	1.98E-06	HHIP		height	20881960	Lango Allen H	Nature	133,653 European ancestry individuals	50,074 European ancestry individuals
					18391952	Weedon M N	Nat Genet	13,665 European ancestry individuals	16,482 European ancestry individuals
					25282103	Wood AR	Nat Genet	253,288 European ancestry individuals	80,067 European ancestry individuals
					18391950	Lettre G	Nat Genet	15,821 European ancestry individuals	17,801 European ancestry individuals
					20546612	Zhao J	BMC Med Genet	8,184 children of European ancestry	NA
			GYPA	height	30595370	Kichaev G	Am J Hum Gene	458,000 European ancestry individuals	NA
6	3.45E-07	GRM4		height	30595370	Kichaev G	Am J Hum Gene	458,000 European ancestry individuals	NA
		HMGA1, MIR6835		height	20881960	Lango Allen H	Nature	133,653 European ancestry individuals	50,074 European ancestry individuals
					31562340	Akiyama M	Nat Commun	159,095 Japanese ancestry individuals	32,692 Japanese ancestry individuals
					20546612	Zhao J	BMC Med Genet	8,184 children of European ancestry	NA
		C6orf1, NUDT3		height	19893584	Kim JJ	J Hum Genet	8,842 Korean ancestry individuals	NA
					28270201	Nagy R	Genome Med	19,920 British ancestry individuals from 6863 families.	NA
		RPS10, RPS10−NUDT3		height	31562340	Akiyama M	Nat Commun	159,095 Japanese ancestry individuals	32,692 Japanese ancestry individuals
					30595370	Kichaev G	Am J Hum Gene	458,000 European ancestry individuals	NA
		PACSIN1		height	30595370	Kichaev G	Am J Hum Gene	458,000 European ancestry individuals	NA
6	8.53E-09	UHRF1BP1, TAF11		height	30595370	Kichaev G	Am J Hum Gene	458,000 European ancestry individuals	NA
		ANKS1A, TCP11, SCUBE3, ZNF76		height	18391951	Gudbjartsson DF	Nat Genet	30,968 European ancestry individuals	8,541 European ancestry individuals
					20546612	Zhao J	BMC Med Genet	8,184 children of European ancestry	NA
			C6orf106	height	25282103	Wood AR	Nat Genet	253,288 European ancestry individuals	80,067 European ancestry individuals
			SPDEF	height	28552196	Tachmazidou I	Am J Hum Genet	53,588 European ancestry individuals	205,003 European ancestry individuals
			PACSIN1	height	30595370	Kichaev G	Am J Hum Gene	458,000 European ancestry individuals	NA
6	1.21E-05	FKBP5	RPL10A	height	25282103	Wood AR	Nat Genet	253,288 European ancestry individuals	80,067 European ancestry individuals

		ARMC12, SRPK1, SLC26A8, MAPK14, MAPK13		height	30595370	Kichaev G	Am J Hum Gene	458,000 European ancestry individuals	NA
			TEAD3, TULP1	height	31562340	Akiyama M	Nat Commun	159,095 Japanese ancestry individuals	32,692 Japanese ancestry individuals
			PPARD	height	21998595	N'Diaye A	PLoS Genet	20,427 African ancestry individuals	16,436 African American individuals
6	9.02E-06	BCKDHB		height	25282103	Wood AR	Nat Genet	253,288 European ancestry individuals	80,067 European ancestry individuals
6	5.63E-06	CENPW		height	25282103	Wood AR	Nat Genet	253,288 European ancestry individuals	80,067 European ancestry individuals
		RSPO3		height	28270201	Nagy R	Genome Med	19,920 British ancestry individuals from 6863 families.	NA
7	4.25E-05	GATAD1		height	18391951	Gudbjartsson DF	Nat Genet	30,968 European ancestry individuals	8,541 European ancestry individuals
					20546612	Zhao J	BMC Med Genet	8,184 children of European ancestry	NA
		PEX1		height	25282103	Wood AR	Nat Genet	253,288 European ancestry individuals	80,067 European ancestry individuals
					20546612	Zhao J	BMC Med Genet	8,184 children of European ancestry	NA
		CDK6, FAM133B	KRIT1	height	25282103	Wood AR	Nat Genet	253,288 European ancestry individuals	80,067 European ancestry individuals
					18391952	Weedon M N	Nat Genet	13,665 European ancestry individuals	16,482 European ancestry individuals
					20546612	Zhao J	BMC Med Genet	8,184 children of European ancestry	NA
			ANKIB1	height	30595370	Kichaev G	Am J Hum Gene	458,000 European ancestry individuals	NA
13	1.06E-05	DLEU1	DLEU7, DLEU7−AS1	height	28552196	Tachmazidou I	Am J Hum Genet	53,588 European ancestry individuals	205,003 European ancestry individuals
					25282103	Wood AR	Nat Genet	253,288 European ancestry individuals	80,067 European ancestry individuals
					18391952	Weedon M N	Nat Genet	13,665 European ancestry individuals	16,482 European ancestry individuals
					18391951	Gudbjartsson DF	Nat Genet	30,968 European ancestry individuals	8,541 European ancestry individuals
					20546612	Zhao J	BMC Med Genet	8,184 children of European ancestry	NA
					20881960	Lango Allen H	Nature	133,653 European ancestry individuals	50,074 European ancestry individuals
			RNASEH2B−AS1, RNASEH2B	height	30595370	Kichaev G	Am J Hum Gene	458,000 European ancestry individuals	NA
15	7.39E-07	ADAMTSL3, SH3GL3		height	18391951	Gudbjartsson DF	Nat Genet	30,968 European ancestry individuals	8,541 European ancestry individuals
					25282103	Wood AR	Nat Genet	253,288 European ancestry individuals	80,067 European ancestry individuals
					18391952	Weedon M N	Nat Genet	13,665 European ancestry individuals	16,482 European ancestry individuals
					20546612	Zhao J	BMC Med Genet	8,184 children of European ancestry	NA

15	1.15E-05	ACAN	MRPS11, HAPLN3, ISG20, MRPL46	height	20881960	Lango Allen H	Nature	133,653 European ancestry individuals	50,074 European ancestry individuals
					25282103	Wood AR	Nat Genet	253,288 European ancestry individuals	80,067 European ancestry individuals
					18391952	Weedon M N	Nat Genet	13,665 European ancestry individuals	16,482 European ancestry individuals
					21998595	N'Diaye A	PLoS Genet	20,427 African ancestry individuals	16,436 African American individuals
					20546612	Zhao J	BMC Med Genet	8,184 children of European ancestry	NA
					28552196	Tachmazidou I	Am J Hum Genet	53,588 European ancestry individuals	205,003 European ancestry individuals
			FANCI, RLBP1	height	25282103	Wood AR	Nat Genet	253,288 European ancestry individuals	80,067 European ancestry individuals
			DET1, AEN	height	25282103	Wood AR	Nat Genet	253,288 European ancestry individuals	80,067 European ancestry individuals
					30595370	Kichaev G	Am J Hum Gene	458,000 European ancestry individuals	NA
18	2.39E-08	CABLES1		height	23563607	Berndt SI	Nat Genet	8,097 European ancestry tall individuals, 8,099 European ancestry short individuals	4,872 European ancestry tall individuals, 4,831 European ancestry short individuals
					18391951	Gudbjartsson DF	Nat Genet	30,968 European ancestry individuals	8,541 European ancestry individuals
					20881960	Lango Allen H	Nature	133,653 European ancestry individuals	50,074 European ancestry individuals
					20546612	Zhao J	BMC Med Genet	8,184 children of European ancestry	NA
20	1.03E-12	UQCC1, GDF5	FAM83C−AS1, FAM83C, CPNE1, RBM12, NFS1, RBM39, ROMO1, MYH7B, TRPC4AP	height	18391951	Gudbjartsson DF	Nat Genet	30,968 European ancestry individuals	8,541 European ancestry individuals
					18391952	Weedon M N	Nat Genet	13,665 European ancestry individuals	16,482 European ancestry individuals
					28270201	Nagy R	Genome Med	19,920 British ancestry individuals from 6863 families.	NA
					18193045	Sanna S	Nat Genet	6669 European ancestry individuals	3860 African American or Afro-Caribbean 23684 European ancestry individuals
					31562340	Akiyama M	Nat Commun	159,095 Japanese ancestry individuals	32,692 Japanese ancestry individuals
					30595370	Kichaev G	Am J Hum Gene	458,000 European ancestry individuals	NA
					25429064	He M	Hum Mol Genet	36,227 East Asian ancestry individuals	57,699 East Asian ancestry individuals
		CEP250		height	18391951	Gudbjartsson DF	Nat Genet	30,968 European ancestry individuals	8,541 European ancestry individuals
			MMP24, EIF6	height	18391951	Gudbjartsson DF	Nat Genet	30,968 European ancestry individuals	8,541 European ancestry individuals
			ERGIC3, FER1L4	height	28552196	Tachmazidou I	Am J Hum Genet	53,588 European ancestry individuals	205,003 European ancestry individuals
			PHF20	height	25282103	Wood AR	Nat Genet	253,288 European ancestry individuals	80,067 European ancestry individuals
			PROCR	height	23563607	Berndt SI	Nat Genet	8,097 European ancestry tall individuals, 8,099 European ancestry short individuals	4,872 European ancestry tall individuals, 4,831 European ancestry short individuals
			EDEM2	height	25282103	Wood AR	Nat Genet	253,288 European ancestry individuals	80,067 European ancestry individuals

20	7.91E-08	CEP250, ERGIC3, FER1L4, CPNE1, RBM12, NFS1, ROMO1, RBM39	GDF5	height	18391951	Gudbjartsson DF	Nat Genet	30,968 European ancestry individuals	8,541 European ancestry individuals
					28552196	Tachmazidou I	Am J Hum Genet	53,588 European ancestry individuals	205,003 European ancestry individuals
					31562340	Akiyama M	Nat Commun	159,095 Japanese ancestry individuals	32,692 Japanese ancestry individuals
					18391952	Weedon M N	Nat Genet	13,665 European ancestry individuals	16,482 European ancestry individuals
		PHF20, CNBD2		height	25282103	Wood AR	Nat Genet	253,288 European ancestry individuals	80,067 European ancestry individuals
		EPB41L1		height	23563607	Berndt SI	Nat Genet	8,097 European ancestry tall individuals, 8,099 European ancestry short individuals	4,872 European ancestry tall individuals, 4,831 European ancestry short individuals
Haplotype bases regional GREML model									
6	1.96E-05	SAMD5		height	30595370	Kichaev G	Am J Hum Gene	458,000 European ancestry individuals	NA

Supplementary Table 2. Top genomic regions identified by SNP/ haplotype-based model for MDD. The columns are chromosome number, the association p-values, the genes within region, genes within 400kb of regions, associated trait, and a list of studies reporting gene association with trait.

SNP based regional GREML model

CHR	P value	Genes	Genes within 400kb	Traits	Reference Study				
					PMID	First author	Journal	Discovery sample (size and ancestry)	Replication sample (size and ancestry)
13	2.41E-05		DACH1	depressive symptom measurement, stressful life event measurement	30718454	Arnau-Soler A	Transl Psychiatry	4919 European ancestry individuals	NA
				age at onset of Alzheimer's disease	26830138	Herold C	Mol Psychiatry	3524 European ancestry individuals	NA
				brain volume measurement	31676860	Zhao B	Nat Genet	19629 European ancestry individuals	NA
				schizophrenia, attempted suicide	31164008	Mullins N	Am J Psychiatry	8764 European ancestry individuals	NA

Haplotype bases regional GREML model

CHR	P value	Genes	Genes within 400kb	Traits	PMID	First author	Journal	Discovery sample (size and ancestry)	Replication sample (size and ancestry)
18	2.30E-06		DCC	unipolar depression	29700475	Wray NR	Nat Genet	480359 European ancestry individuals	NA
					30718901	Howard DM	Nat Neurosci	446238 European ancestry individuals	1306354 European ancestry individuals
					27422368	Zeng Y	Biol Psychiatry	21,387 European ancestry individuals	18,759 European ancestry individuals
				self-reported educational attainment, mathematical ability, cognitive function measurement	29186694	Lam M	Cell Rep	436124 European ancestry individuals	NA
					33414549	Demange PA	Nat Genet	257700 European ancestry individuals	NA
					27225129	Okbay A	Nature	405072 European ancestry individuals	NA
					30038396	Lee JJ	Nat Genet	1131438 European ancestry individuals	NA
				Neuroticism measurement	29292387	Turley P	Nat Genet	168105 European ancestry individuals	NA
					30643256	Baselmans BML	Nat Genet	523783 European ancestry individuals	59206 European ancestry individuals
				brain volume measurement		Hibar DP	Nature	13171 European ancestry individuals	736 Hispanic or Latin American<br>15130 European<br>545 East Asian

3	5.19E-06	MYRIP	EIF1B−AS1	Alzheimer's disease	20197096	Stein JL	Neuroimage	742 European ancestry individuals	NA
				sleep measurement	17903308	Gottlieb DJ	BMC Med Genet	738 NR	NA
				reading and spelling ability	30741946	Gialluisi A	Transl Psychiatry	2,563 European ancestry individuals	NA
				information processing speed	21130836	Luciano M	Biol Psychol	4,039 European ancestry individuals	NA
				Grey matter density measurement	31530798	Alliey-Rodriguez N	Transl Psychiatry	483 European ancestry, 44 NR, 250 African American or Afro-Caribbean ancestry	NA
				white matter hyperintensity measurement	32517579	Armstrong NJ	Stroke	16,832 European ancestry individuals, 736 African American individuals, 658 Hispanic individuals	8,428 European and unknown ancestry individuals
			MOBP	Alzheimer's disease (cognitive decline)	23535033	Sherva R	Alzheimers Dement	303 European ancestry cases	NA
				Cognitive performance	19734545	Need AC	Hum Mol Genet	958 European ancestry, 207 East Asian ancestry, 26 NR, 104 South Asian ancestry	NA
				Progressive supranuclear palsy	21685912	Höglinger GU	Nat Genet	4027 European ancestry, 45 NR, 329 Other	4611 European ancestry individuals
				Attention deficit hyperactivity disorder	18839057	Lesch KP	J Neural Transm	593 European ancestry individuals	NA
2	2.07E-05	ANO7	PASK, MTERFD2, SNED1	Unipolar depression, schizophrenia, bipolar disorder, concentration dose ratio, response to antipsychotic drug	25944848	Athanasiu L	J Psychopharmacol	829 European ancestry cases	NA
			BOK, THAP4	brain measurement	20171287	Stein JL	Neuroimage	740 European ancestry individuals	NA
			PPP1R7	PHF-tau measurement	32450446	Wang H	Neurobiol Aging	1285 NR	NA
1	2.46E-05	KCNK1	KIAA1804	age at onset Alzheimer's disease,	26830138	Herold C	Mol Psychiatry	3524 European ancestry individuals	NA
				cognitive function measurement	28808816	Xu C	Biogerontology	278 East Asian ancestry individuals	NA
12	2.70E-05	AMIGO2, PCED1B		Alzheimer's disease	19118814	Beecham GW	Am J Hum Genet	988 European ancestry individuals	458 European ancestry individuals
				PHF-tau measurement	32450446	Wang H	Neurobiol Aging	1285 NR	NA
				bipolar disorder, attempted suicide	31164008	Mullins N	Am J Psychiatry	8764 European ancestry individuals	NA

10	2.82E-05		NRP1	unipolar depression, alcohol dependence	29071344	Zhou H	JAMA Psychiatry	3,041 African American individuals, 1,618 European ancestry individuals	1,612 African American individuals, 1,551 European ancestry individuals
				schizophrenia, response to paliperidone, schizophrenia symptom severity measurement	27846195	Li Q	Pharmacogenet Genomics	1,390 European ancestry cases	NA
				migraine disorder	23793025	Anttila V	Nat Genet	118710 European ancestry individuals	NA
				brain measurement	32665545	van der Meer D	Nat Commun	26,502 European ancestry individuals	NA
			LINC00838	Schizophrenia	21682944	Alkelai A	Int J Neuropsychopharmacol	331 European ancestry individuals	189 Greater Middle Eastern ancestry individuals
2	4.59E-05		HNRNPA3, MIR4444−2, MIR4444−1, NFE2L2, MIR3128, LOC100130691, MIR6512	alcohol consumption measurement	30643251	Liu M	Nat Genet	1,039,210 European ancestry individuals	NA
2	4.67E-05	CRIM1	FEZ2	cognitive function measurement, mathematical ability	30038396	Lee JJ	Nat Genet	1131438 European ancestry individuals	NA
				intelligence	29844566	Davies G	Nat Commun	300,486 European ancestry individuals	NA
			VIT	brain volume measurement	31676860	Zhao B	Nat Genet	19629 European ancestry individuals	NA
